# Patent Medicines in Atlanta

**Published:** 1883-11

**Authors:** 


### PATENT MEDICINES IN ATLANTA.

A paragraph was reproduced in a leading journal of this city, a few days ago, which seemed to make it a subject for exultation that our city had become a great and conspicuous center for the manufacture, advertising and wholesale vending of patent medicines.

If this claim is true, and we are not at all disposed to doubt it, is it a subject that should elicit the commendations of any patriot or right-minded citizen? Does the mere fact that there is money—immense profits in this miserable business of making and selling patent drugs, make it right—sanctify it in the eye of humanity and virtue?

In the name of all that is good, is there not already here in Atlanta, too much of that truckling, soulless, utilitarian spirit of covetousnesss, which is ready to justify and accept any and everything just short of theft and direct murder, only so it is largely profitable to the producers or venders? The end justifies the means, say they, by their acts, if not in so many words.

It is remarkable that the exceedingly profitable results of this unprofessional business, to say the very least of it, has not only stimulated the unscrupulous greed which sustains and feeds it, but contrary to reason and expectation, it actually enhances the very credulity of the ignorant masses on which these vampires fatten.

Let me quote on this point, from a pertinent article by Wm. L. Turner, of Philadelphia, in the *American Journal of Pharmacy*: "The well-known fact that almost fabulous wealth has been accumulated in this field of enterprise, and even in some, yea many cases, upon a single preparation, would appear to indicate that the percentage of profit on this class of preparations, has been to the manufacturer and proprietor enormously large, which, instead of producing upon the public mind abundant and conclusive evidence of their comparative worthlessness has rather tended to increase the credulity of the deceived as well as the wealth of the deceiver. The amount sold and the expenditure of money in effecting its sale, is heralded as an indication of merit by those who push them, as though they were the disinterested parties, who expended their time and substance for the alleviation of the ills of life, and the exclusive benefit of their fellows."

When we read the paragraph referred to at the beginning of this paper, we confess to a tingle of shame that our city should be thus published to the world as a great centre of patent drug manufacture. If this be a notable sign of her prosperity, then, indeed, fortunes in the money sense are being built up among her business men, but at the fearful expenditure of a power infinitely more precious than wealth, and by the perpetration of unspeakable wrong upon a too confiding people and country.

In another paper on this pregnant subject we design to pay due regard to certain religious journals and Gospel ministers, who seem to have no scruples in lending the great authority of their names and influence to this same enormity of deception upon communities, whose well-being they are under the most binding of all obligations to watch over and preserve.

